# Radical Observations about People’s Teeth, No. II

**Published:** 1874-01

**Authors:** 


### RADICAL OBSERVATIONS ABOUT PEOPLE'S MOUTHS AND TEETH. NO. II.

To the Editor of the Dental Register:

Having in a former communication said something about the class of people negligent and careless about their teeth, I shall take a cursory view of those who think enough about personal appearance and comfort to give attention to the most noticeable feature of the face—the mouth. I shall take it for granted that there is no one nowadays ignorant enough to deny the positive good in dentistry, or that would not avail themselves of its benefits and advantages, provided they had means to do so. Proceeding, then, from this recognition of fact, I will consider dentistry as divided into two kinds, good and bad, or the cheap kind and the well paid kind, and without any attemps at apology or explanation claim that the latter is the cheaper at the beginning and at the end. And please to bear in mind that I look at the subject purely from an outside point of view—the point of an observer merely.

I can conceive, then, that an operation of any kind, by either a good dentist or a bad one, costs the patient: first, time; second, physical discomfort, if not positive pain; and third, money. He must sit in the operator's chair all the time necessary to complete the work, endure all the horrid sensations peculiar to it, and pay for it when it is done. If he is a man of good observation, that is, if he looks beneath the surface of things, among which will be a close comparison of the results and the mere pretentions of dental advertisers, he will soon be able to discriminate between the good and the bad, and conclude that the best results remain to men who promise little and perform much.

He will soon learn that there is no more virtue in a cheap piece of dentistry than in the patent medicines speciously advertised and assured as infallible remedies for whole alphabets of diseases. He will learn that the good dentist brings to each individual case the treatment and the work peculiarly adapted to it, just as the good physician—the true physician—treats his patient according to his age, condition in life, temperament ard symptoms.

The little instrument which most resembles the human voice is old, perhaps, as the human race. It is the first a boy begins to learn, and the last that any body masters, and there is a steady demand for—the flute. There are factories devoted to its manufacture; yet it is very seldom a perfect instrument is found. There is only one maker in the world who is recognised as producing the most faultless flute, and his success lies in the fact that he makes and finishes with his own hands every instrument that bears the impress of his name. He does not sell you a pipe that has gone through a mill, carrying with it all the imperfections of illy-seasoned timber and badly fitted joints; but permits to leave his establishment that instrument only which has stood the test of his critical judgment, into which enters the experience of eye and ear long-used to look for perfection in mechanical construction, and faultlessness of harmony.

The curse of latter-day dentistry is factory-made dentures sets of teeth made in so-called steam dental establishments. The idea associated with steam in the public mind is rapidity and cheapness. When I was a child, I used to wonder at the labor expended in the manufacture of sparrow shot. It was inconceivable by me how a man could make those suggestive little pellets so round and bright and uniform. I saw a sculptor at work, but felt no respect for him or his labor; the shot maker was the wonderful man I wanted to see. Since I came to look at this subject of dentistry, I have compared my youthful experience with the propensity of the majority of people who purchase artificial teeth, and find that they go together by the common law of affinity.

People become terribly exercised about their eyes or their ears, and travel hundreds of miles and expend hundreds of dollars to consult a specialist; but if a "job" of filling is needed or an artificial "set" must be inserted, the cheapest man—of course—is the best, and nine cases out of ten, he is the man that gets the work. But it is not to be wondered at; for when the nice model sets are shown them by the cheap knaves who have the hardihood to advertise themselves as doctors of dental surgery, and promise a complete set of artificial teeth for ten dollars, they are deceived into the belief that the model set and the set about to be ordered will correspond in beauty, beyond which they do not see. They do not consider the adaptability of either the workmanship or the material used to their individual case, nor is it intended by the quack dentist that they shall. To get a new set of teeth in the briefest possible time, at the cheapest possible price, is the prime object, and the devil take the rest.

I think it entirely possible that a genius can make good music with a factory-made flute, but I have to remark that great musicians invariably use fine instruments. I will not deny that there are some fine sets of teeth made in the dental factories, and that occasionally they are found to fit and give tolerable satisfaction, but they are the exception and not the rule, the result of chance and not of intelligent design and skillfully adapted labor.

A great painter, famous for the wonderful perfection of his colors, was asked once what he mixed them with. "Mix them with," he replied, "brains, sir, with brains." That sort of mixing is not done by the cheap dentist ; if it were, there would not be so many foul-mouthed rubber teeth, grinning ghastly in the public face, nor such horrid hot mush attempts at speaking by men and women who should know better than impose themselves upon society with such repulsive, though invariable, accompaniments of bad dentistry. E. B.

